# 1206. Effects of Climatic Variability on Lyme Disease Outbreaks in California

**DOI:** 10.1093/ofid/ofab466.1398

**Published:** 2021-12-04

**Authors:** Raghesh Varot Kangath, Rajasree Pai Ramachandra, Buddhika Madurapperuma, Luke Scaroni

**Affiliations:** 1 VA Medical Center, California, San Francisco, California; 2 San Francisco VA Medical Center, San Francisco, California; 3 Humboldt State University, Arcata, California

## Abstract

**Background:**

Climate change has increased the risk of tick borne infections. The life cycle and prevalence of deer ticks are strongly influenced by temperature. Warmer temperatures associated with climate change are projected to increase the range of suitable tick habitats driving the spread of Lyme disease (LD). Short winters could also increase tick activity increasing the risk of exposure. This study examines the relationship between LD incidence and temperature-precipitation and their anomalies in CA counties.

**Methods:**

Trends and relationships of Lyme Disease (LD) cases and climatic factors were analyzed among the California counties from 2000 to 2019. Lyme disease tabulate data and climatic data were obtained from Centers for Disease Control, and NOAA, and Climate Data Guide respectively. Canonical correspondence analysis (CCA) was performed using variables: (i) LD cases, (ii) precipitation & anomaly, and temperature & anomaly. The CCA ordination explained the variability between LD cases and climatic variables. Biplots were used to visualize the associations between LD cases and climatic anomalies.

**Results:**

We compared the countywide LD cases in relation to climatic factors in California from 2000 to 2019. A total of 96 cases in 2000, 117 cases in 2009, and 144 cases in 2019 were reported in the 55 counties of California. Santa Clara reported the highest LD cases in 2003 (23 cases; 16%), followed by Los Angeles in 2013 (20 cases; 18%) and Santa Cruz in 2017 (19 cases; 13%). CCA ordination showed distinguishable clustering patterns between southern California counties (Santa Clara, Santa Cruz, Alameda, and San Diego) and northern coast and Klamath mountains range (Humboldt, Trinity, Shasta, and Siskiyou) regions (Fig. 1). Moderate mean annual temperature (56.5 °F - 62.5 °F) and temperature anomaly (3.8 °F - 5.5 °F) were the most important variable predictor for high LD outbreak.

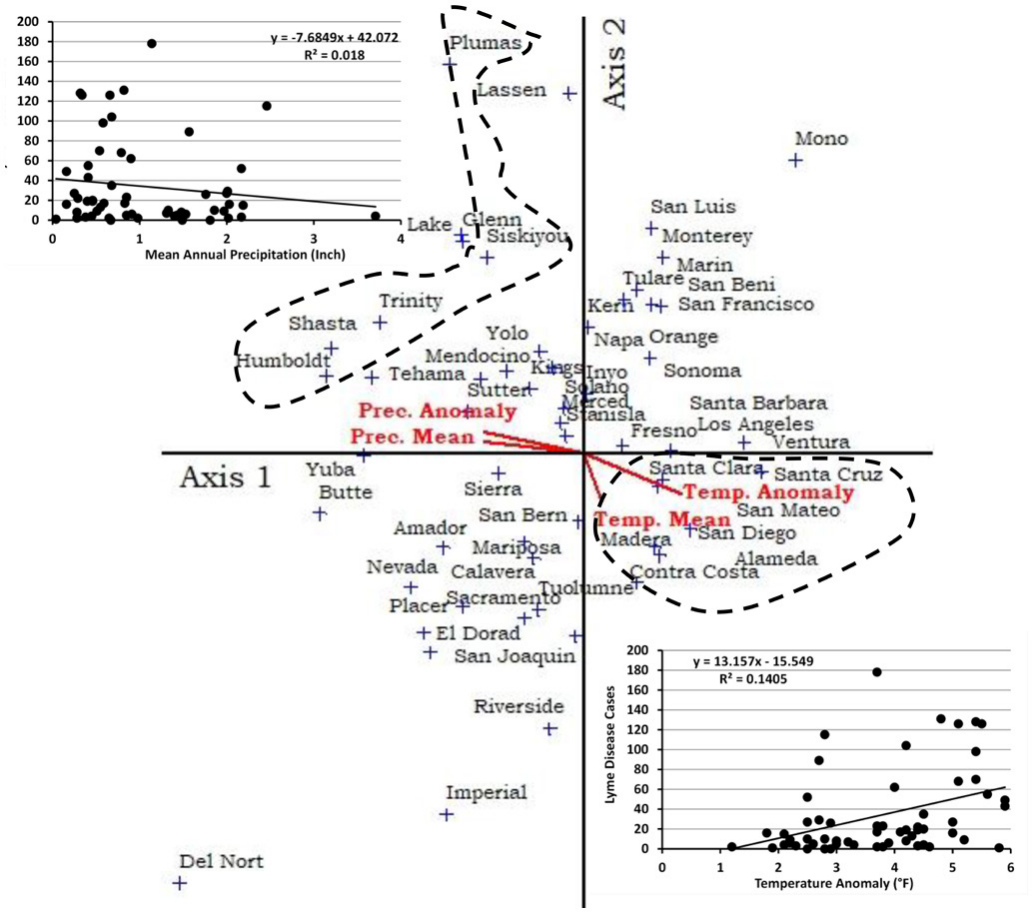

The CCA ordination shows the relationships between Lyme Disease and climatic variables for the 55 Counties of California. The bottom right circle represents Lyme cases positively correlated with temperature anomaly (3.8 °F - 5.5 °F) and moderate annual mean temperature (56.5 °F - 62.5 °F). The upper left circle represents Lyme cases negatively correlated with mean annual precipitation.

**Conclusion:**

Moderate temperature with low moist spell anomalies in the south neighboring CA counties showed a positive influence on LD outbreak. The climatic conditions in those areas suitable for Oak trees and masting acorn resulting in the establishment of tick and host (deer) populations. We recommend robust surveillance and lab testing for patients with a history of tick bites in these regions.

**Disclosures:**

**All Authors**: No reported disclosures

